# IL-10 Enhances Human Natural Killer Cell Effector Functions via Metabolic Reprogramming Regulated by mTORC1 Signaling

**DOI:** 10.3389/fimmu.2021.619195

**Published:** 2021-02-23

**Authors:** Zixi Wang, Di Guan, Jianxin Huo, Subhra K. Biswas, Yuhan Huang, Yuansheng Yang, Shengli Xu, Kong-Peng Lam

**Affiliations:** ^1^Department of Microbiology and Immunology, Yong Loo Lin School of Medicine, National University of Singapore, Singapore, Singapore; ^2^Bioprocessing Technology Institute, A*STAR (Agency for Science, Technology and Research), Singapore, Singapore; ^3^NUS Graduate School for Integrative Sciences & Engineering (NGS), National University of Singapore, Singapore, Singapore; ^4^Singapore Immunology Network (SIgN), A*STAR (Agency for Science, Technology and Research), Singapore, Singapore; ^5^Department of Physiology, Yong Loo Lin School of Medicine, National University of Singapore, Singapore, Singapore

**Keywords:** NK cell, IL-10, cytotoxicity, IFN-γ, mTOR, metabolism

## Abstract

Cell metabolism plays a pivotal role in regulating the effector functions of immune cells. Stimulatory cytokines, such as interleukin (IL)-2 or IL-12 and IL-15, activate glycolysis and oxidative phosphorylation in natural killer (NK) cells to support their enhanced effector functions. IL-10, a pleiotropic cytokine, is known to suppress macrophage activation but stimulate NK cells. However, it remains unclear if IL-10 has an effect on the metabolism of human NK cells and if so, what metabolic mechanisms are affected, and how these metabolic changes are regulated and contribute to the effector functions of NK cells. In this study, we demonstrate that IL-10 upregulates both glycolysis and oxidative phosphorylation in human NK cells, and these metabolic changes are crucial for the enhanced effector functions of NK cells. Mechanistically, we unravel that IL-10 activates the mammalian target of rapamycin complex 1 (mTORC1) to regulate metabolic reprogramming in human NK cells.

## Introduction

Natural killer (NK) cells are innate cytotoxic lymphocytes that target tumor and virus-infected cells to prevent malignancy and systemic infection (1, 2). Unlike cytotoxic T cells, NK cells exert immediate cytotoxicity without prior priming and kill tumor cells in an antigen-independent manner. Therefore, NK cells can be used as off-the-shelf therapeutics to treat cancer patients without the induction of graft-versus-host disease, making them an attractive modality in cell-based cancer immunotherapy (3, 4). The activation of NK cells is regulated by the engagement of NK cell receptors (NKRs) and cytokines (1). NK cells express both activating (e.g., NKG2D and CD16) and inhibitory (e.g., NKG2A) NKRs, which recognize corresponding ligands on target cells and transduce either stimulatory or inhibitory signals (5). The overall balance of stimulatory and inhibitory signals determines whether NK cells are activated or inhibited (6). Cytokines also play critical roles in regulating NK cell activation. Stimulatory cytokines, such as interleukin (IL)-2, IL-12 and IL-15, promote proliferation and activation of NK cells (7), while inhibitory cytokines, e.g., transforming growth factor β (TGF-β), inhibit NK cell maturation, proliferation and functions (8, 9).

IL-10 is an important cytokine known to regulate the function of many immune cell types (10). It was initially identified as a cytokine secreted by T helper 2 cells (11). Later, many other leukocytes, including macrophages, T, B, NK, and dendritic cells, were also shown to produce IL-10 (12, 13). IL-10 is a pleiotropic cytokine that exerts multifaceted effects on various immune cells. It exhibits anti-inflammatory effects on monocytes, macrophages, dendritic cells and T helper cells to suppress their functions, such as production of pro-inflammatory cytokines, phagocytosis and antigen presentation, thereby preventing tissue damage and maintaining immune system homeostasis (14–17). On the other hand, IL-10 is shown to exert stimulatory effects on cytotoxic T and NK cells. For example, IL-10 enhances the anti-tumor activity of cytotoxic T cells both *in vitro* and *in vivo* (18, 19). IL-10 also increases the expression of activation and cytotoxicity-related genes in NK cells and promotes tumor lysis by NK cells (20, 21). When combined with IL-15 or IL-18, IL-10 enhances the proliferation, cytotoxicity and cytokine production of NK cells (22, 23). Moreover, the cytomegalovirus analog of human IL-10 produced during human cytomegalovirus infection promotes antibody-dependent cell-mediated cytotoxicity (ADCC) and NK cell cytotoxicity by enhancing the expression of stimulatory NKRs on NK cells (24).

It is well-appreciated that cell metabolism is critical for driving the response of immune cells to environmental stimuli (25). Previous studies demonstrated that resting NK cells rely on oxidative phosphorylation (OXPHOS) for their basal activity (26), and IL-2- or IL-12/IL-15-stimulated NK cells upregulate glycolysis and OXPHOS to support their effector functions (26, 27). NK cells also upregulate glycolysis and OXPHOS to support their cytokine production and cytotoxicity upon engagement of the activating NKRs (28). In contrast, TGF-β inhibits NK cell proliferation and effector functions induced by IL-15 or IL-2, and this is coupled with the suppression of NK cell metabolism (9, 29). Recently, IL-10 was demonstrated to suppresses LPS-induced activation of macrophages and inhibit their metabolic reprogramming by dampening glycolysis (30). In this study, we examined if IL-10 regulates NK cell metabolism and how IL-10-induced metabolic changes, if any, affect NK cell effector functions. We found that IL-10 treatment upregulates glycolysis and OXPHOS in human NK cells, and these metabolic changes lead to increased interferon (IFN)-γ production and cytotoxicity. We further demonstrated that mammalian target of rapamycin complex 1 (mTORC1) signaling is required for IL-10-induced metabolic reprogramming and augmented effector functions of human NK cells.

## Materials and Methods

### Antibodies and Reagents

Anti-CD16 antibody (clone 3G8) was purchased from BioLegend. BUV395-CD3 (clone UCHT1), PE-Cy™7-CD56 (clone B159), BV421-CD210a (clone 3F9), BV421-IgG2a (clone R35-95), FITC-CD107a (clone H4A3), PE-FasL (clone NOK-1) were purchase from BD Biosciences. Antibodies used for western blot were as follows: mouse anti-β-actin was purchased from Santa Cruz Biotechnology. Rabbit anti-phospho-S6 ribosomal protein (Ser235-Ser236) antibody, rabbit anti-phospho-p70 S6 kinase (Thr389) antibody, rabbit anti-p70 S6 kinase antibody, and mouse anti-phospho-STAT3 (Tyr705) antibody were purchased from Cell Signaling Technology.

Human IL-10 was purchased from Miltenyi Biotec, and human recombinant IL-2 was from R&D System. Cell-Tak™ Cell and Tissue Adhesive was purchased from Corning. Rapamycin and CellTrace Violet were from Invitrogen. Ficoll® Paque Plus, 2-deoxyglucose (2-DG), etomoxir, UK5099, BPTES, oligomycin, FCCP, rotenone, antimycin and concanamycin A were purchased from Sigma-Aldrich. Human IFN-γ ELISA MAX™ Standard Set was from BioLegend. Human Granzyme B ELISA development kit was from Mabtech. Lactate Assay Kit was from Sigma-Aldrich. Fixable viability dye eFluor 780 (FVD) was purchased from eBioscience. 2-NBDG was purchased from Life Technologies. Stattic was from Santa Cruz Biotechnology. SuperSignal West Pico/Dura chemiluminescent substrate was from Pierce.

### Purification and *ex vivo* Expansion of Human NK Cells

Human peripheral blood was obtained from healthy donors with written consent. Ethics was granted by the Institutional Review Board of Singapore Immunology Network (SIgN) (201306-04). Peripheral blood mononuclear cells (PBMCs) were isolated from blood using Ficoll® Paque Plus by gradient centrifugation. NK cells were enriched from human PBMCs by negative selection using EasySep™ Human NK Cell Isolation Kit as per manufacturer's instructions. Isolated NK cells were cultured in BIOTARGET™ medium supplemented with 10% fetal bovine serum (FBS, Biowest). For some experiments, NK cells were expanded as previously described (28). Briefly, human PBMCs were co-cultured with irradiated K562 feeder cells that were engineered to express membrane-bound (mb)-IL-15, mb-IL-21 and 4-1BB ligand in BIOTARGET™ medium supplemented with 10% FBS and human recombinant IL-2 (50 IU/ml). After 14 days of culture, NK cells were specifically enriched (the purity of CD56^+^ CD3^−^cells > 90%) and significantly expanded in number and used for the subsequent analyses.

### NK Cell Treatment

NK cells were treated with human IL-10 (50 ng/ml) in a humidified incubator at 37°C (5% CO_2_) for 16 h. To test the dose-dependent effects of IL-10, NK cells were treated with IL-10 at 5, 20, 50, and 100 ng/ml or left untreated. The supernatant of IL-10 stimulated and unstimulated NK cells were collected to assess IFN-γ and granzyme B production. NK cells were harvested after stimulation and washed with PBS. To inhibit cell metabolism, IL-10-stimulated NK cells were treated with 2-DG (30 mM) to inhibit glycolysis, oligomycin (2.5 μM) plus the combination of rotenone (500 nM) and antimycin (500 nM) to inhibit OXPHOS, etomoxir (150 μM) to inhibit fatty acid oxidation, UK5099 (10 μM) to inhibit pyruvate transportation, or BPTES (5 μM) to inhibit glutaminolysis in the presence of IL-10 for 3 and 6 h for cytotoxicity analysis and ELISA, respectively. To inhibit mTORC1 activity, NK cells were treated with IL-10 and rapamycin (20 nM) for 1, 3, 4, or 6 h for western blot or 16 h for ELISA, cytotoxicity assay and metabolic analysis. When indicated, NK cells were treated with IL-10 and rapamycin at 0, 0.5, 5, 20 50 nM for 16 h for ELISA and cytotoxicity assay. To inhibit STAT3 activity, NK cells were treated with IL-10 and Stattic (50 μM) for 6 h before western blotting. To inhibit degranulation, NK cells were stimulated with IL-10 for 16 h before treated with 1 or 5 nM concanamycin A for 3 h. To engage CD16 on NK cells, the anti-CD16 antibody (15 μg/ml) was coated on 24-well plates at 4°C overnight. Untreated and IL-10-stimulated NK cells were placed on the coated plate and incubated at 37°C (5% CO_2_) for 4 h. When indicated, NK cells with various treatments described above were co-cultured with K562 cells at an effector to target (E:T) ratio of 1:1.5 for 1.5 h. The supernatant was collected for ELISA, and the cells were analyzed on a LSR II flow cytometer (BD Biosciences).

### Analysis of Cell Metabolism

NK cells were treated with IL-10 in the presence or absence of rapamycin for 16 h as indicated. Real-time analyses of ECAR and OCR of treated and non-treated NK cells were performed using XF-24 Extracellular Flux Analyzer (Seahorse Bioscience) as described previously (31). Briefly, NK cells were resuspended in XF base and assay medium (Agilent Technologies) for ECAR and OCR analysis, respectively. Cells were adhered to an XF 96-well-microplate (Seahorse Bioscience) coated with Cell-Tak™ Cell and Tissue Adhesive at a concentration of 200,000 cells per well. Cells were then starved in a non-CO_2_ incubator at 37°C for 1 h to block glycolysis. ECAR was measured under basal conditions followed by consecutive addition of glucose (10 mM) to induce glycolysis, oligomycin (1 μM) to inhibit ATP production in OXPHOS, and 2-DG (100 mM) to inhibit glycolysis. Glycolysis was calculated as the difference of ECAR before and after glucose injection. Glycolytic capacity of NK cells was quantified by the difference of ECAR before and after the injection of 2-DG. OCR of NK cells was measured under basal conditions followed by the sequential treatment of oligomycin (1 μM), FCCP (1 μM) and the combination of rotenone (500 nM) and antimycin (500 nM) (R&A). OCR after the addition of R&A represents non-mitochondrial respiration. When indicated, etomoxir (150 μM), UK5099 (10 μM), or BPTES (5 μM) was injected before the addition of oligomycin. Basal respiration was calculated as the difference between OCR before the addition of oligomycin and non-mitochondrial respiration. ATP-linked respiration was quantified as the difference of OCR before and after the addition of oligomycin. The changes of OCR before and after the addition of R&A represents maximal respiration.

### Lactate Detection

NK cells were stimulated with IL-10 or left unstimulated in a humidified incubator at 37°C (5% CO_2_) for 16 h. Culture supernatant was collected and assayed for lactate detection using Lactate Assay Kit according to the manufacturer's instructions.

### ELISA

NK cells were stimulated with IL-10 and subjected to various treatments as described above. The supernatant of NK cells with or without treatment was collected and assayed for IFN-γ and granzyme B secretion using ELISA as described previously (28).

### Cytotoxic Assay

K562 target cells were labeled with CellTrace Violet (1 μM) for 10 min in PBS at 37°C (5% CO_2_). NK cells were stimulated with IL-10 for 16 h or left unstimulated at 37°C (5% CO_2_). NK cells were then collected and co-cultured with CellTrace Violet-labeled K562 cells at E:T ratios of 0.5:1, 1:1 and 1.5:1 for *ex vivo* expanded NK cells or of 2:1 and 5:1 for freshly-isolated NK cells. When indicated, IL-10 stimulated NK cells were treated with 2-DG or oligomycin plus rotenone and antimycin or both as described above. NK cells after treatment were collected and washed with PBS twice to remove metabolic inhibitors before co-cultured with K562 cells for 1.5 h. 2-DG was added back to the co-culture in groups with glycolysis inhibition. To inhibit mTORC1 activity in NK cells, NK cells were treated with IL-10 and rapamycin for 16 h. NK cells were then washed with PBS and subsequently co-cultured with K562 cells as described above. After incubation, cells were harvested and stained with FVD in PBS for 10 min on ice followed by flow cytometry analysis for dead cell detection. The percentage of K562 cells killed by NK cells was calculated using the following equation: $\%\;Killing=\frac{\%Dead\;K562_{\left(E:T\;ratio=n:1\right)}-\%Dead\;K562_{\left(E:T\;ratio=0:1\right)}}{100\%-\%Dead\;K562_{\left(E:T\;ratio=0:1\right)}}$.

### Flow Cytometry Analysis

To detect CD107a expression, NK cells were stimulated with IL-10 and treated with various metabolic inhibitors as described above. NK cells with or without various treatments were stimulated with plate-bound anti-CD16 antibody for 4 h or with CellTrace Violet labeled-K562 cells at an E:T ratio of 1.5:1 for 1.5 h. For glucose uptake assay, NK cells were treated with IL-10 for 16 h or left untreated in a humidified incubator at 37°C (5% CO_2_). Untreated and treated NK cells were cultured in glucose-free RPMI 1640 medium supplemented with 10% FBS and 2-NBDG (30 μM) in a humidified incubator (37°C, 5% CO_2_) for 1 h. Untreated NK cells without 2-NBDG incubation served as the negative control. Cells were then collected and stained with a saturating concentration of antibodies for 20–30 min on ice. NK cells were identified as CD56^+^ CD3^−^ cells.

### Western Blotting

NK cells were stimulated with IL-10 for 1, 3, 4, 6, and 16 h as indicated. To inhibit the mTORC1 and STAT3 pathway, freshly-isolated NK cells were stimulated with IL-10 and rapamycin (20 nM) or IL-10 and Stattic (50 μM), respectively, for 6h. Western blotting was performed as described previously (32). Briefly, NK cells were harvested and lysed with ice-cold lysis buffer (10 mM Tris-HCl, pH 8.0, 150 mM NaCl, 1 mM EDTA, 1% Igepal CA-630, 0.2 mM Na3VO4) supplemented with a Protease Inhibitor Cocktail (Roche). Equal amounts of proteins were loaded onto SDS-PAGE for protein separation and subsequently transferred onto polyvinylidene difluoride membranes. The membranes were blotted for target proteins by incubating with specific primary antibodies and secondary antibodies conjugated with horseradish peroxidase. Membranes were then washed and developed with a SuperSignal West Pico/Dura chemiluminescent substrate.

### Statistical Analysis

Statistical analysis was conducted using GraphPad Prism 8. Two-tailed Student's *t*-test was used to compare the means of 2 groups. One-way ANOVA tests were used to compare means for more than 2 groups, with Dunnett's tests used for multiple comparisons. *P*-value < 0.05 was considered as significant. ^*^P < 0.05; ^**^P < 0.01; ^***^P < 0.001.

## Results

### IL-10 Increases IFN-γ Production and Cytotoxicity of NK Cells

Previous studies mainly explored the role of IL-10 on murine NK cells or NK92 cells, either alone or in combined with other stimulatory cytokines, such as IL-15 and IL-18 (21–23). In this study, we first aimed to clarify the effect of IL-10 on human NK cells that were either *ex vivo* expanded or freshly purified from human PBMCs. For *ex vivo* expansion of NK cells, human PBMCs were co-cultured with IL-2 and K562 cells expressing membrane-bound (mb)-IL-15, mb-IL-21, and 4-1BB ligand as described previously (28). We found that the *ex vivo* expanded and freshly-isolated NK cells expressed comparable levels of IL-10 receptor (IL-10R) on their cell surfaces, as assessed by flow cytometric analysis (Supplementary Figure 1).

**Figure 1:**
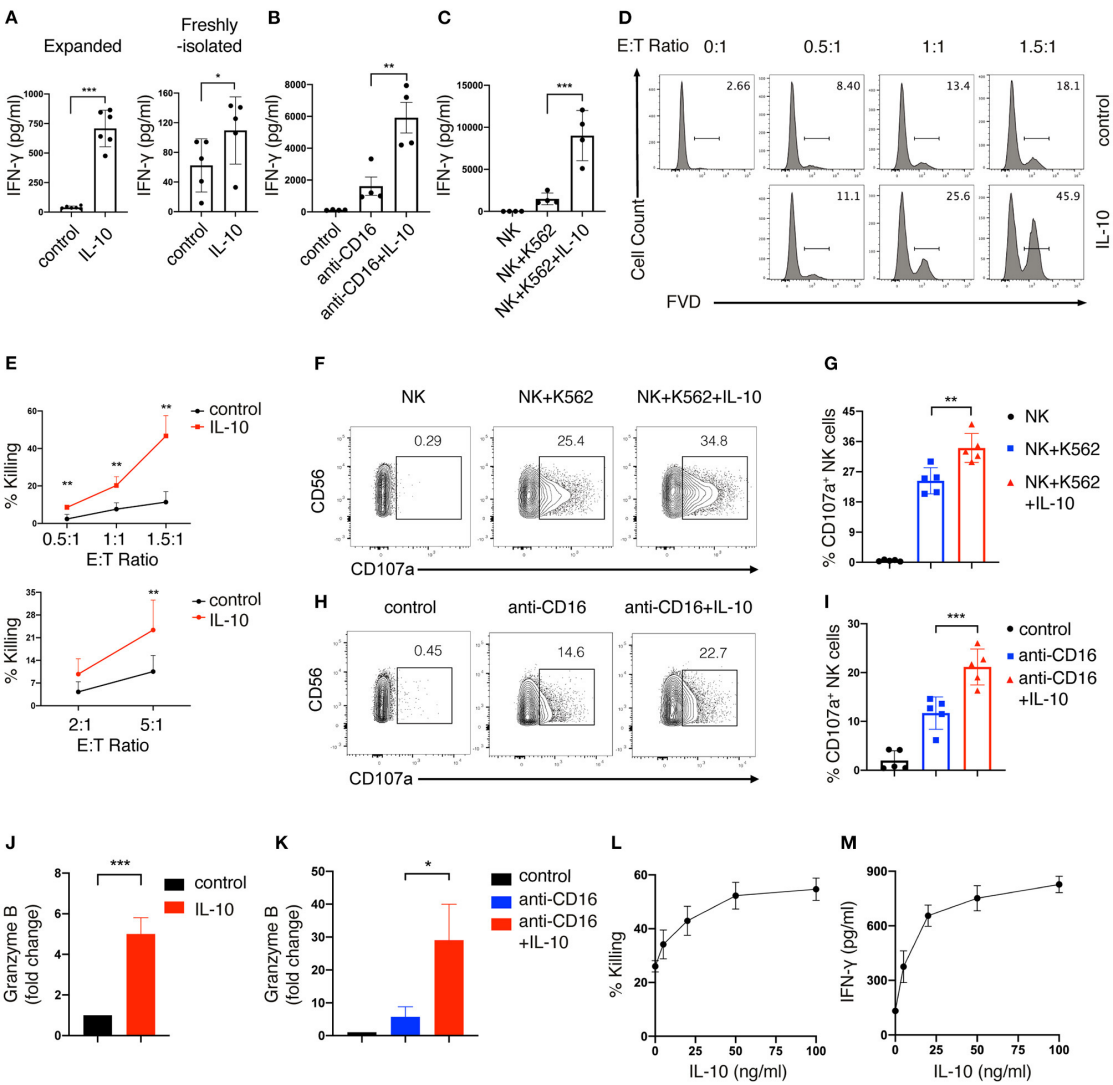
IL-10 enhances IFN-γ secretion and cytotoxicity of human NK cells. **(A–C)** IFN-γ secretion of NK cells induced by IL-10. *Ex vivo* expanded (**A**, left panel) and freshly-isolated **(A**, right panel**)** NK cells were stimulated with IL-10 (50 ng/ml) for 16 h. *Ex vivo* expanded NK cells treated with or without IL-10 were also stimulated with anti-CD16 antibody for 4 h **(B)** or co-cultured with K562 cells for 2 h **(C)**, respectively. Culture supernatants were collected and IFN-γ was measured using ELISA. NK cells cultured without stimulation served as control. **(D,E)** Killing of K562 cells by NK cells with or without IL-10 stimulation. NK cells were cultured with or without IL-10 for 16 h. The pre-treated NK cells were washed with PBS before co-culturing with CellTrace Violet labeled-K562 cells for 1.5 h at various E:T ratios as indicated. Dead K562 cells were determined by flow cytometry analysis of cells stained with Fixable Viability Dyes (FVD). The percentages of dead K562 cells after co-culturing with *ex vivo* expanded NK cells were shown in the histogram **(D)**. The percentage of K562 cells killing by *ex vivo* expanded **(E**, upper panel**)** and freshly-isolated **(E**, lower panel**)** NK cells was quantified. **(F,G)** K562 cell-induced expression of CD107a on NK cells with or without IL-10 stimulation. NK cells were treated with IL-10 for 16 h before co-culturing with K562 cells at an E:T ratio of 1.5:1 for 1.5 h. Expression of CD107a on NK cells were examined by flow cytometry. The percentage of CD107a expressing NK cells was quantified **(G)**. **(H,I)** Anti-CD16 antibody-induced expression of CD107a on NK cells with or without IL-10 stimulation. NK cells were cultured with IL-10 for 16 h or left untreated and subsequently stimulated with anti-CD16 antibody for 4 h. The expression of CD107a on NK cells were examined using flow cytometry. **(J,K)** Granzyme B secretion of NK cells with or without IL-10 stimulation. NK cells were treated with or without IL-10 for 16 h followed by another 4 h of culture in the absence **(J)** or presence **(K)** of anti-CD16 antibody. The supernatant was collected after stimulation, and granzyme B was assessed by ELISA. **(L,M)** Killing of K562 cells **(L)** and IFN-γ secretion **(M)** by NK cells treated with IL-10 of different concentrations. NK cells were left untreated or treated with IL-10 of 5, 20, 50, and 100 ng/ml for 16 h. Culture supernatant was collected and assayed for IFN-γ detection using ELISA. NK cells were co-cultured with K562 cells for 1.5 h at an E:T ratio of 1.5:1. Untreated NK cells served as control. FACS data were representative of multiple experiments. Each dot in the bar charts represents one experiment. The results were presented as Mean ± SD [*n* = 4–6 for **(A–C)**, *n* = 4 for **(E,K)**, *n* = 5 for **(G,I,J)**, n=2 for **(L,M)**]. Data were analyzed using independent Student's *t*-test for **(A,E,J)**, and one-way ANOVA with Dunnett's test for **(B,C,G,I,K)**. *P < 0.05; **P < 0.01; ***P < 0.001.

Next, we determined the effect of IL-10 treatment on the effector functions of human NK cells. We first measured the secretion of IFN-γ by NK cells after IL-10 treatment using ELISA. We found that IFN-γ production was significantly elevated by ~ 18-fold in expanded NK cells upon IL-10 treatment (700 pg/ml) compared with unstimulated cells (40 pg/ml) (Figure 1A, left panel). IL-10 also showed a stimulatory but less potent effect on the secretion of IFN-γ by freshly-isolated NK cells (Figure 1A, right panel). We further examined the effect of IL-10 on IFN-γ secretion by NK cells that were either treated with anti-CD16 antibody to engage the Fcγ receptor to mimic ADCC or co-cultured with target K562 cells. We observed that the treatment with anti-CD16 antibody (Figure 1B) and co-culture with K562 cells (Figure 1C) induced NK cells to increase IFN-γ production to 1,600 and 1,400 pg/ml, respectively. Strikingly, the addition of IL-10 further elevated the IFN-γ secretion of these stimulated NK cells to 6,000 pg/ml (Figure 1B) and 9,000 pg/ml (Figure 1C). These data together suggest that IL-10 enhances the production of IFN-γ in human NK cells.

We also assessed the effect of IL-10 on the cytotoxicity of NK cells. *Ex vivo* expanded NK cells were first treated with or without IL-10 for 16 h before co-culturing with K562 cells at various E:T (effector: target) ratios. The extent of cytotoxicity was measured by flow cytometry. We observed that IL-10 treatment significantly enhanced the cytotoxicity of *ex vivo* expanded NK cells, as shown by the enhanced killing of K562 cells by NK cells at various E:T ratios (Figures 1D,E, upper panel). Similarly, freshly-isolated NK cells also exhibited increased killing of K562 cells upon the addition of IL-10 (Figure 1E, lower panel). These data suggest that IL-10 can augment NK cell cytotoxicity. We proceeded to investigate the mechanisms contributing to the enhanced cytotoxicity of IL-10-stimulated NK cells. Co-culture with K562 cells (Figures 1F,G) or treatment with anti-CD16 antibody (Figures 1H,I) upregulated the expression of CD107a, a surrogate for the activation of granule exocytosis, which was significantly increased by IL-10 treatment in NK cells. The treatment of IL-10 also significantly increased the production of granzyme B in NK cells when they were cultured in the absence (Figure 1J) or presence (Figure 1K) of anti-CD16 antibodies. Inhibiting degranulation using concanamycin A significantly dampened the cytotoxicity of IL-10 stimulated NK cells against K562 cells (Supplementary Figure 2). These results indicate that IL-10 increases the cytotoxicity of NK cells by enhancing degranulation. We also examined the dose-dependent effects of IL-10 on NK cell cytotoxicity and cytokine secretion. We observed that NK cell cytotoxicity (Figure 1L) and IFN-γ secretion (Figure 1M) were significantly enhanced as IL-10 concentration increases from 5 to 50 ng/ml, which were not further elevated when IL-10 concentration reached 100 ng/ml. These data collectively suggest that IL-10 exerts stimulatory effects on human NK cells and enhances their effector functions.

### IL-10 Upregulates Glycolysis and OXPHOS in NK Cells

**Figure 2:**
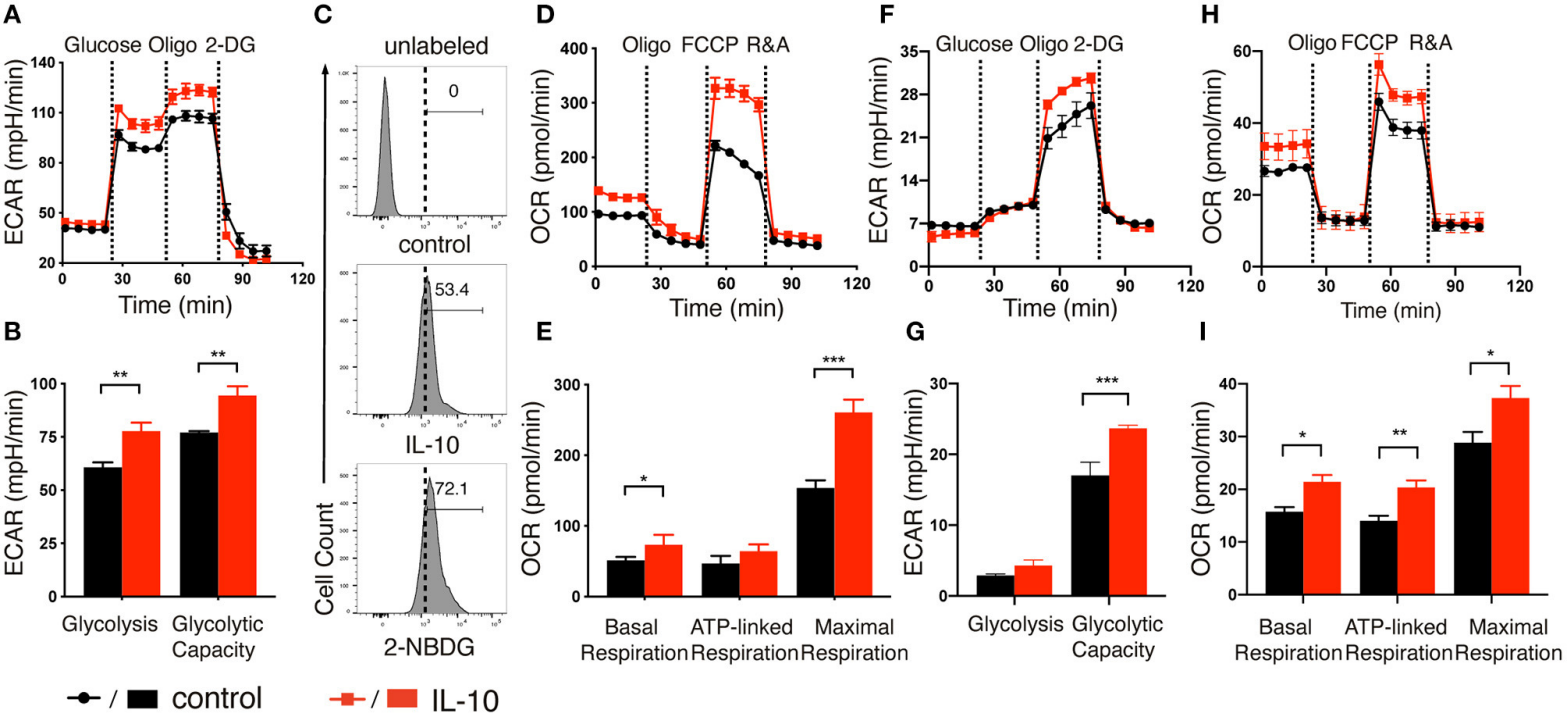
IL-10 induced metabolic changes in NK cells. **(A,F)** Real-time analyses of extracellular acidification rate (ECAR) of *ex vivo* expanded **(A)** and freshly-isolated **(F)** NK cells with (red) or without (black) IL-10 stimulation for 16 h. ECAR of NK cells were measured with sequential addition of glucose (10 mM), ATP synthase inhibitor oligomycin (oligo) (1 μM), and non-metabolizable glucose analog 2-deoxyglucose (2-DG) (100 mM). **(B,G)** Quantification of glycolysis and glycolytic capacity of *ex vivo* expanded **(B)** and freshly-isolated **(G)** NK cells. **(C)** The uptake of glucose by the *ex vivo* expanded NK cells with or without IL-10 stimulation. NK cells with or without IL-10 treatment were incubated with the fluorescent glucose analog 2-NBDG for 30 min. Untreated NK cells not incubated with 2-NBDG (denoted unlabeled) served as negative control. **(D,H)** Real-time analyses of oxygen consumption rate (OCR) of *ex vivo* expanded **(D)** and freshly-isolated **(H)** NK cells treated with (red) or without (black) IL-10. OCR of NK cells were measured with consecutive addition of oligomycin (1 μM), the mitochondrial uncoupler FCCP (500 nM), and inhibitors of mitochondrial electron transport chain complex I and III, rotenone (500 nM) and antimycin (500 nM) (R&A). **(E,I)** Quantification of basal respiration, ATP-linked respiration and maximal respiration of *ex vivo* expanded **(E)** and freshly-isolated **(I)** NK cells. The results were presented as Mean ± SEM [*n* = 4–5 for **(A,B,D–I)**]. Data were compared using independent Student's *t*-test. *P < 0.05; **P < 0.01; ***P < 0.001.

After demonstrating the stimulatory effects of IL-10 on NK cells, we next explored if IL-10 would affect the metabolism of NK cells, as studies had shown that metabolic reprogramming affects NK cell effector functions (26–29), and IL-10 was demonstrated to suppress the LPS-induced activation of macrophage through metabolic reprogramming (30). To this end, we stimulated *ex vivo* expanded NK cells with IL-10 for 16 h and measured their extracellular acidification rate (ECAR) and oxygen consumption rate (OCR), which allowed for direct quantification of glycolysis and OXPHOS in these cells. We found that treatment with IL-10 increased the ECAR level in NK cells (Figure 2A). Both glycolysis and glycolytic capacity of NK cells were significantly increased upon IL-10 stimulation (Figure 2B). Consistent with their heightened glycolysis and glycolytic capacity, IL-10-stimulated NK cells displayed increased glucose intake compared to the unstimulated cells, as demonstrated by their augmented uptake of fluorescence-labeled glucose analog 2-NBDG (Figure 2C). Consistent with the increased ECAR, IL-10-stimulated NK cells also produced more lactate than unstimulated cells (Supplementary Figure 3). Moreover, IL-10 stimulation also increased OCR levels in NK cells (Figure 2D). Specifically, the basal respiration, ATP-linked respiration and maximal respiration of NK cells were all increased in NK cells upon IL-10 treatment (Figure 2E). Together, our data indicate that IL-10 upregulates glycolysis and OXPHOS in human NK cells.

Because *ex vivo* expanded NK cells were cultured in the presence of IL-2 and feeder cells that expressed mb-IL-15, mb-IL-21, and 4-1BB ligand, it is possible that these factors could interfere with IL-10-induced metabolic changes. To address this concern, we examined the ECAR and OCR of freshly-isolated NK cells after IL-10 treatment. We observed that freshly-isolated NK cells exhibited a relatively lower glycolysis rate with or without IL-10 treatment compared with *ex vivo* expanded NK cells (Figures 2F,G). However, when ATP production in OXPHOS was inhibited by oligomycin, the ECAR level was drastically increased to meet the metabolic requirement of NK cells (Figures 2F,G), which was not so obvious in the *ex vivo* expanded NK cells (Figures 2A,B). Interestingly, IL-10 treatment led to a significant increase in the glycolytic capacity of freshly-isolated NK cells (Figure 2G), similar to that seen in *ex vivo* expanded NK cells (Figure 2B). When we examined OCR levels in the freshly-isolated NK cells treated with IL-10, it was evident that the basal respiration, ATP-linked respiration and maximal respiration were all enhanced in these cells compared to unstimulated control (Figures 2H,I). These results together suggest that IL-10 induces metabolic reprogramming in NK cells by augmenting their glycolysis and OXPHOS.

**Figure 3:**
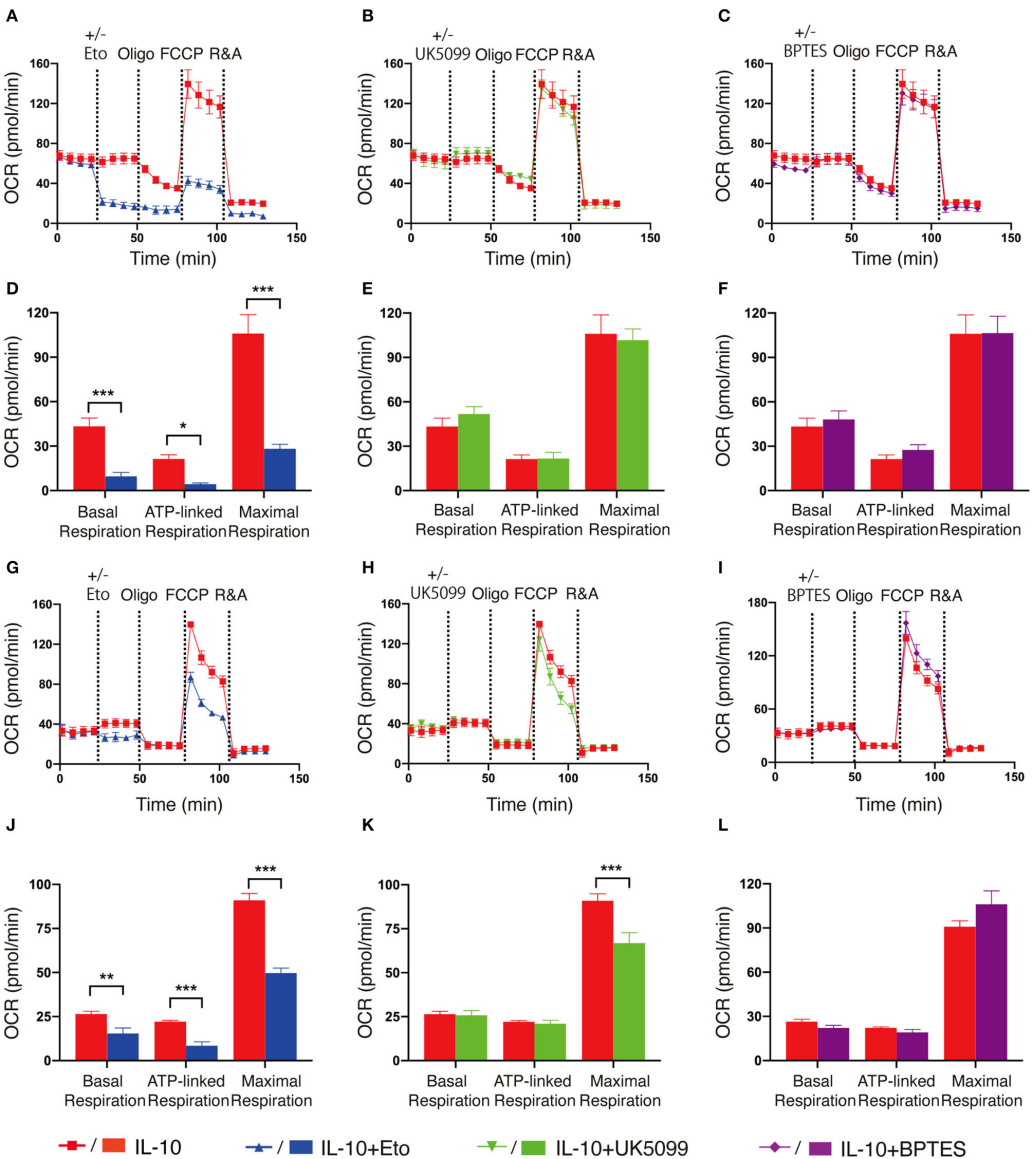
Fatty acid oxidation is the main source of elevated OXPHOS in IL-10 stimulated NK cells. *Ex vivo* expanded and freshly-isolated NK cells were treated with IL-10 for 16 h. **(A–C)** Real-time analyses of OCR in IL-10 stimulated *ex vivo* expanded NK cells with inhibition of fatty acid oxidation **(A)**, pyruvate transportation **(B)**, or glutaminolysis **(C)**. OCR was measured at baseline followed by the addition of medium (red) or etomoxir (Eto, 150 μM, blue, **A**) or UK5099 (10 μM, green, **B**), or BPTES (5 μM, purple, **C**) and the sequential injection of oligomycin, FCCP, and rotenone plus antimycin (R&A). **(D–F)** Quantification of basal respiration, ATP-linked respiration and maximal respiration of IL-10 stimulated *ex vivo* expanded NK cells with inhibition of fatty acid oxidation **(D)**, pyruvate transportation **(E)**, or glutaminolysis **(F)**. **(G–I)** Real-time analyses of OCR of IL-10 stimulated freshly-isolated NK cells with inhibition of fatty acid oxidation **(G)**, pyruvate import **(H)**, or glutaminolysis **(I)**. OCR was measured as described in **(A–C)**. **(J–L)** Quantification of basal respiration, ATP-linked respiration and maximal respiration of IL-10 stimulated freshly-isolated NK cells with inhibition of fatty acid oxidation **(J)**, pyruvate transportation **(K)**, or glutaminolysis **(L)**. The results were presented as Mean ± SEM [*n* = 4-5 for **(A–F)**, *n* = 3–6 for **(G–L)**]. Data were compared using independent Student's *t*-test. *P < 0.05; **P < 0.01; ***P < 0.001.

As fatty acid oxidation (FAO), glycolysis and metabolism of amino acids such as glutamine are all able to fuel the TCA cycle and contribute to OXPHOS, we next determined the main contributing source of the elevated OXPHOS in IL-10 stimulated NK cells. We inhibited FAO, pyruvate transportation, and glutamine metabolism using etomoxir, UK5099 and BPTES, respectively, and examined their effects on OXPHOS of IL-10 stimulated NK cells. We observed that inhibiting FAO significantly suppressed the basal, ATP-linked and maximal respiration of IL-10 stimulated NK cells (Figures 3A,D), whereas inhibiting pyruvate transportation (Figures 3B,E) and glutaminolysis (Figures 3C,F) had little impact on OXPHOS. These results indicate that FAO is the primary source of OXPHOS in *ex vivo* expanded NK cells stimulated with IL-10. We also examined the contribution of the different metabolic pathways to OXPHOS in freshly-isolated NK cells. Similarly, inhibiting FAO also drastically impaired OXPHOS in freshly-isolated NK cells stimulated by IL-10 (Figures 3G,J). Interestingly, suppressing pyruvate import also decreased the maximal respiration of freshly-isolated NK cells treated with IL-10 (Figures 3H,K), whereas inhibiting glutaminolysis has no impact on OXPHOS (Figures 3I,L). These results suggest that FAO is the main source for OXPHOS in both *ex vivo* expanded and freshly-isolated NK cells stimulated with IL-10. Glycolysis also partially contributed to the elevated OXPHOS in freshly-isolated NK cells.

### IL-10-Induced Metabolic Changes Enhance NK Cell Effector Functions

**Figure 4:**
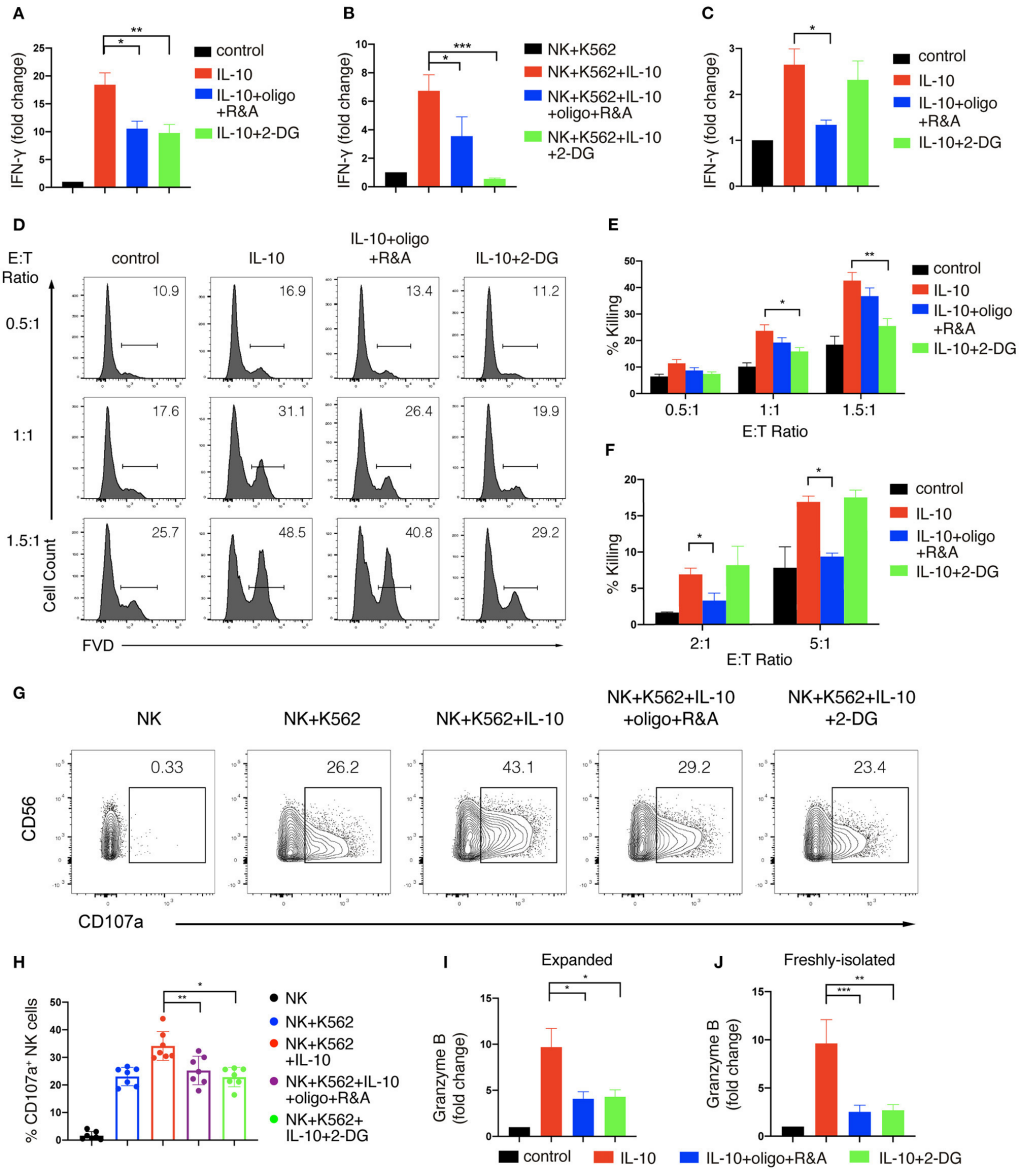
Glycolysis and OXPHOS are required for the enhanced effector functions of IL-10 treated NK cells. **(A–C)** IFN-γ secretion of NK cells treated with IL-10 and metabolic inhibitors. NK cells were pre-treated with IL-10 for 16 h and washed with PBS. The pre-treated *ex vivo* expanded **(A,B)** or freshly-isolated **(C)** NK cells were re-stimulated with IL-10 in the absence **(A,C)** or presence **(B)** of K562 cells for 6 h. 2-DG or oligomycin plus R&A were also added during the last 6 h to inhibit glycolysis or OXPHOS. Supernatant was collected after stimulation and IFN-γ was measured using ELISA. Unstimulated NK cell cultures served as control. **(D–F)** Killing of K562 cells by NK cells treated with IL-10 and metabolic inhibitors. NK cells were stimulated with IL-10 for 16 h and subsequently treated with metabolic inhibitors as described above for another 3 h. The pre-treated NK cells were washed with PBS twice before co-cultured with CellTrace Violet-labeled K562 cells at various E:T ratios as indicated. 2-DG was added back to the co-culture of groups with glycolysis inhibition. Untreated NK cells which were co-cultured with K562 cells served as control. Dead K562 cells were determined by flow cytometry analysis of cells stained with FVD, and the percentages of dead K562 cells were shown in the histogram **(D)**. The percentage of K562 cells killing by *ex vivo* expanded **(E)** and freshly-isolated **(F)** NK cells was quantified. **(G,H)** CD107a expression on NK cells treated with IL-10 and metabolic inhibitors. *Ex vivo* expanded NK cells were stimulated with IL-10 for 16 h before incubated with 2-DG or oligomycin plus R&A for 3 h. The unstimulated and pre-treated NK cells were co-cultured with K562 cells at an E:T ratio of 1.5:1 for 1.5 h. The expression of CD107a **(G)** on NK cells was examined by flow cytometry. The percentages of CD107a **(H)** expressing NK cells after various stimulation as indicated were quantified. Each dot represented a single experiment **(H)**. **(I,J)** Granzyme B secretion of NK cells treated with IL-10 and metabolic inhibitors. *Ex vivo* expanded **(I)** and freshly-isolated **(J)** NK cells were treated as described in **(A,C)**. Supernatant was collected after stimulation, and granzyme B was detected using ELISA. Unstimulated NK cell culture served as control. The results were presented as Mean ± SEM [*n* = 7 for **(A,E,H)**, *n* = 4 for **(B)**, *n* = 5 for **(C)**, *n* = 2 for **(F)**, *n* = 6 for **(I)**, *n* = 3 for **(J)**]. Samples were compared using One-way ANOVA with Dunnett's test. *P < 0.05, **P < 0.01, ***P < 0.001.

We next asked if IL-10-induced increase in glycolysis and OXPHOS contributed to the enhanced effector functions of NK cells. To this end, we first determined the impact of metabolic changes on NK cell production of IFN-γ upon IL-10 treatment. We used 2-DG, a non-metabolizable analog of glucose, to inhibit glycolysis and oligomycin together with rotenone and antimycin, the inhibitors of ATP synthase and electron transport chain complex I and III, to suppress OXPHOS. We found that IL-10-induced IFN-γ secretion by *ex vivo* expanded NK cells was decreased from ~20- to ~10-fold when either glycolysis or OXPHOS was suppressed (Figure 4A). When NK cells co-cultured with K562 cells were examined, we found that inhibition of glycolysis or OXPHOS also suppressed IFN-γ secretion of NK cells induced by IL-10, with the suppression of glycolysis having a more drastic impact (Figure 4B). Next, we examined the effect of glycolysis or OXPHOS inhibition on IL-10-induced IFN-γ secretion by freshly-isolated NK cells. We found that IL-10 induced IFN-γ secretion of NK cells was significantly repressed when OXPHOS was inhibited but was not affected when glycolysis was inhibited alone (Figure 4C). These data suggest that IL-10-induced metabolic changes are required to support enhanced IFN-γ secretion of NK cells.

We further examined if enhanced cytotoxicity induced by IL-10 in NK cells was affected by metabolic changes. We first stimulated NK cells with IL-10 for 16 h and cultured them for another 3 h in the presence of various metabolic inhibitors as indicated. Subsequently, the inhibitors were washed away, and the NK cells were co-cultured with K562 cells at different E:T ratios to assess NK cell cytotoxicity, with 2-DG added back to the groups examined for the effect of glycolysis inhibition. Similar to the results shown in Figure 1D, IL-10 treatment increased the killing of K562 cells by *ex vivo* expanded NK cells at various E:T ratios (Figures 4D,E). IL-10-enhanced NK cell cytotoxicity was significantly suppressed by 2-DG treatment but was slightly decreased when OXPHOS was inhibited, suggesting that glycolysis is more important than OXPHOS in supporting IL-10-induced NK cell cytotoxicity. Interestingly, we noticed some differences in the metabolic requirements to support IL-10-induced cell cytotoxicity between *ex vivo* expanded and freshly-isolated NK cells. We found that the inhibition of OXPHOS instead of glycolysis significantly attenuated IL-10-induced cytotoxicity of freshly-isolated NK cells (Figure 4F). We also demonstrated that the increased upregulation of CD107a on IL-10-stimulated NK cells was significantly diminished by the inhibition of glycolysis or OXPHOS (Figures 4G,H). Consistently, IL-10-induced granzyme B secretion in both *ex vivo* expanded and freshly-isolated NK cells were significantly suppressed by OXPHOS or glycolysis inhibition (Figures 4I,J). As FAO and glycolysis, to a lesser extent, fuel OXPHOS in IL-10 stimulated freshly-isolated NK cells, we further explored how these OXPHOS substrates contribute to NK cell effector functions. We found that inhibiting FAO using etomoxir significantly impaired the secretion of granzyme B by NK cells and their cytotoxicity against K562 cells (Supplementary Figure 4). Inhibiting pyruvate also reduced NK cell cytotoxicity and their secretion of granzyme B. Together, these results suggest that glycolysis and OXPHOS are critical for the elevated IFN-γ production and enhanced cytotoxicity of NK cells induced by IL-10.

### mTORC1 Signaling Is Important for the IL-10-Induced Metabolic Reprogramming of NK Cells

mTORC1 is pivotal in regulating cell metabolism by promoting anabolic synthesis and constraining catabolic metabolism to support cell growth and proliferation (33). Previous studies showed that IL-2/IL-12 and IL-15 activate mTORC1 and trigger metabolic reprogramming in murine NK cells and augment their effector functions (34, 35). Human NK cells were also shown to upregulate glycolysis by activating mTORC1 upon IL-2 treatment (27). Since IL-10 upregulates glycolysis and OXPHOS and enhances the effector functions of human NK cells, we investigated if mTORC1 signaling was triggered by IL-10 and responsible for the metabolic changes and enhanced effector functions of IL-10-stimulated NK cells.

**Figure 5:**
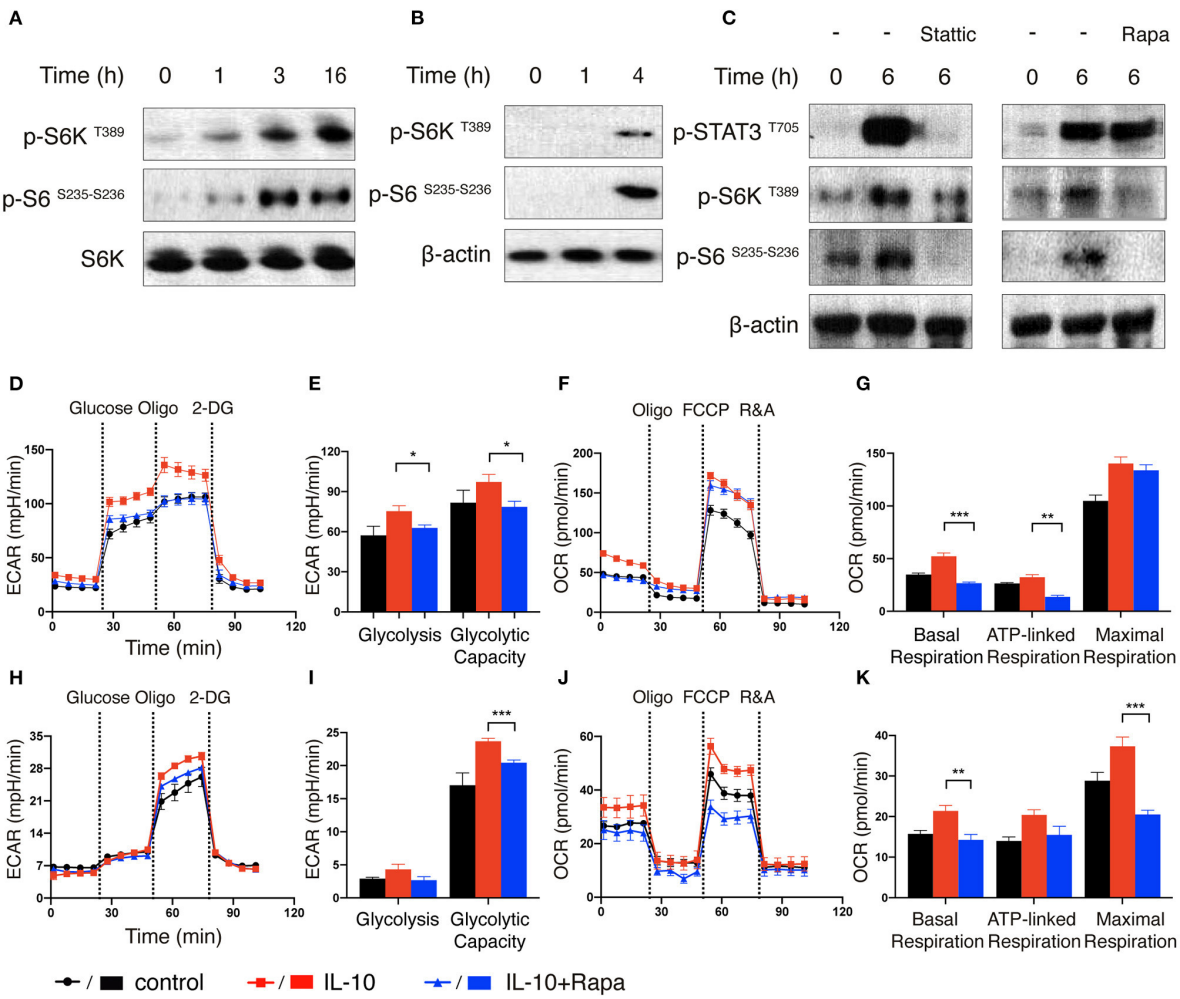
mTORC1 signaling is induced by IL-10 treatment and regulates metabolic reprogramming of NK cells. **(A–C)** Immunoblot analysis of STAT3 and mTORC1 activation in IL-10 stimulated NK cells. *Ex vivo* expanded NK cells were stimulated with IL-10 for 1, 3, and 16 h **(A)**. Freshly-isolated NK cells were stimulated with IL-10 for 1 h and 4 h **(B)** or 6 h **(C)** in the absence or presence of Stattic (50 μM) or rapamycin (Rapa, 20 nM). The expression of p-STAT3 ^T705^, p-S6K ^T389^, p-S6 ^S235−S236^ as well as S6K and β-actin in NK cells were analyzed by western blot. **(D–G)** Real-time analysis of ECAR and OCR of *ex vivo* expanded NK cells with or without the treatment of IL-10 or rapamycin. NK cells were stimulated with IL-10 in the presence or absence of rapamycin for 16 h or left unstimulated. The ECAR **(D)** and OCR **(F)** of *ex vivo* expanded NK cells were analyzed in real time as described in Figure 2. The glycolysis and glycolytic capacity **(E)**, as well as basal respiration, ATP-linked respiration and maximal respiration **(G)** of *ex vivo* expanded NK cells were quantified. **(H–K)** Real-time analysis of ECAR and OCR of freshly-isolated NK cells with or without the treatment of IL-10 or rapamycin. The ECAR **(H)** and OCR **(J)** of NK cells were analyzed in real time. The glycolysis and glycolytic capacity **(I)** as well as basal respiration, ATP-linked respiration and maximal respiration **(K)** of freshly-isolated NK cells were quantified. The results were presented as Mean ± SEM [*n* = 6 for **(D–G)**, *n* = 4-5 for (**H–K)**]. Data were compared using One-way ANOVA with Dunnett's multiple comparison test. *P < 0.05, **P < 0.01, ***P < 0.001.

We first treated *ex vivo* expanded NK cells with IL-10 for different periods of time and examined the phosphorylation status of ribosomal protein S6 kinase (S6K), which is a major downstream target of mTORC1 (36). Compared with unstimulated cells, NK cells treated with IL-10 for 1 h exhibited increased S6K phosphorylation at threonine 389 residue, indicating the activation of S6K (Figure 5A). The phosphorylation of S6K became even more prominent when the NK cells were treated with IL-10 for longer periods (3 or 16 h). Consistent with the increased activation of S6K, we also detected augmented phosphorylation of its substrate ribosomal protein S6 (S6) at serine 235 and 236 residues in IL-10 treated NK cells. Similarly, IL-10 treatment also led to increased phosphorylation of S6K and its substrate S6 in the freshly-isolated NK cells (Figure 5B), suggesting that IL-10 activates mTORC1 in NK cells.

As STAT3 is known to be activated in NK cells upon engagement of IL-10R with IL-10 (10), we next explored if STAT3 was required for the activation of mTORC1 in IL-10 stimulated NK cells. To this end, we treated the freshly-isolated NK cells with IL-10 for 6 h in the absence or presence of Stattic, a STAT3 inhibitor, and compared the activation of mTORC1. As expected, treatment of IL-10 increased STAT3 phosphorylation at tyrosine 705 residue, but this phosphorylation was almost completely abolished in the presence of Stattic (Figure 5C, left panel). Moreover, the inhibition of STAT3 also reduced the phosphorylation of S6K and S6 (Figure 5C, left panel), indicating that STAT3 activation is required for IL-10-triggered mTORC1 signaling in NK cells. On the other hand, when NK cells were treated with IL-10 in the absence or presence of rapamycin, an mTORC1 inhibitor, it was shown that the phosphorylation of STAT3 was mostly unaffected, although rapamycin significantly suppressed the phosphorylation of S6K and S6 (Figure 5C, right panel). These results suggest that STAT3 mediates the activating signals derived from IL-10R and acts upstream of mTORC1.

Next, we determined if mTORC1 signaling is required for IL-10-induced metabolic reprogramming in NK cells. We treated IL-10-stimulated NK cells with rapamycin to inhibit the mTORC1 activity and examined its effect on NK cell metabolism. The elevated glycolysis and glycolytic capacity induced by IL-10 was abolished by rapamycin treatment in *ex vivo* expanded NK cells (Figures 5D,E). Rapamycin treatment also reduced the basal respiration and ATP-linked respiration in IL-10 stimulated NK cells, though it only slightly attenuated the maximal respiration (Figures 5F,G). Likewise, rapamycin inhibition of mTOR signaling also suppressed the increased glycolytic capacity induced by IL-10 in freshly-isolated NK cells (Figures 5H,I). Similarly, the enhanced basal, ATP-linked and maximal respiration were all abolished by rapamycin treatment (Figures 5J,K). These data together suggest that mTORC1 regulates the IL-10-mediated metabolic reprogramming of human NK cells.

### mTORC1 Is Required for IL-10-Enhanced Effector Functions of NK Cells

**Figure 6:**
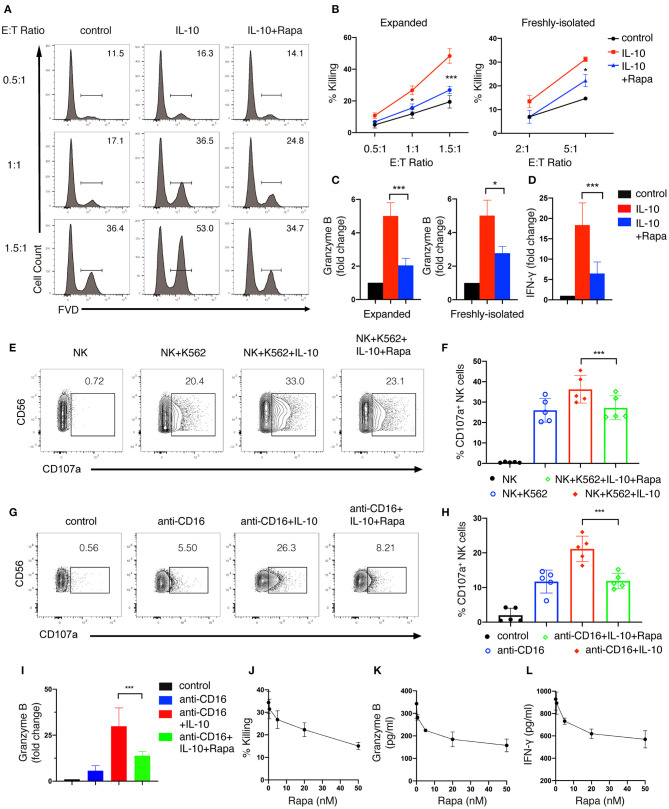
mTORC1 signaling is required for the enhanced effector functions of IL-10 stimulated NK cells. **(A,B)** Killing of K562 target cells by NK cells treated with or without IL-10 and IL-10 with rapamycin. NK cells were untreated or treated with IL-10 in the presence or absence of rapamycin for 16 h. The pre-treated NK cells were washed with PBS twice before co-cultured with K562 cells at various E:T ratios as indicated. The untreated NK cells that co-cultured with K562 cells served as control. Dead K562 cells were determined by flow cytometry analysis of cells stained with FVD, and the percentages of dead K562 cells were shown in the histogram **(A)**. The percentage of K562 cells killing by *ex vivo* expanded **(B**, left panel**)** and freshly-isolated **(B**, right panel**)** NK cells was quantified. **(C)** Granzyme B secretion of *ex vivo* expanded (left panel) and freshly-isolated (right panel) NK cells after treatment of IL-10 with or without rapamycin was examined by ELISA. **(D)** IFN-γ secretion by *ex vivo* expanded NK cells subjected to various treatments as indicated was examined by ELISA. **(E,F)** Examination of K562 target cell-induced expression of CD107a on NK cells with various treatments as indicated. *Ex vivo* expanded NK cells were untreated or treated with IL-10 in the absence or presence of rapamycin. Cells were then washed with PBS and co-cultured with K562 cells at an E:T ratio of 1.5:1 for 1.5 h. The expression of CD107a on NK cells was analyzed by flow cytometry **(E)**. The percentages of CD107a-expressing NK cells was quantified **(F)**. **(G–I)** Examination of anti-CD16 antibody-induced expression of CD107a and granzyme B of NK cells subjected to various treatments as indicated. *Ex vivo* expanded NK cells were untreated or treated with IL-10 or IL-10 with rapamycin as indicated and then stimulated with plate-bound anti-CD16 antibody for 4 h. The expression of CD107a were examined by flow cytometry **(G)**. The percentages of CD107a-expressing NK cells were quantified **(H)**. The supernatant was collected and assayed for ELISA to detect granzyme B **(I)**. **(J–L)** Killing of K562 cells **(J)** and secretion of granzyme B **(K)** and IFN-γ **(L)** by IL-10 stimulated NK cells treated with rapamycin of different concentrations. *Ex vivo* expanded NK cells were treated with IL-10 and rapamycin of 0, 0.5, 5, 20, or 50 nM for 16 h. Culture supernatant was collected and assayed for granzyme B **(K)** and IFN-γ **(L)** detection using ELISA. The pre-treated NK cells were washed twice with PBS and co-cultured with K562 cells for 1.5 h at an E:T ratio of 1.5:1. The percentage of K562 cells killing by NK cells was quantified **(J)**. Results were presented as Mean ± SD [*n* = 5 for **(B**, left panel**)**, *n* = 2 for **(B**, right panel, **J–L)**, *n* = 5 for **(C**, left panel**)**, *n* =4 for **(C**, right panel**)**, *n* = 6 for **(D,I)**, *n* = 5 for **(F, H)**]. Data were analyzed using One-way ANOVA with Dunnett's test. *P < 0.05; ***P < 0.001.

We also examined the effect of inhibiting mTORC1 on IL-10-induced NK cell effector functions. We found that rapamycin treatment significantly suppressed IL-10-induced cytotoxicity in both *ex vivo* expanded (Figures 6A,B, left panel) and freshly-isolated NK cells (Figure 6B, right panel). Meanwhile, the IL-10 induced granzyme B secretion of both *ex vivo* expanded and freshly-isolated NK cells was also inhibited by rapamycin treatment (Figure 6C). Furthermore, IL-10-induced increase in IFN-γ secretion of *ex vivo* expanded NK cells was markedly suppressed by rapamycin (Figure 6D). Consistent with the inhibitory effect on the cytotoxicity of IL-10-treated NK cells, rapamycin treatment also significantly attenuated IL-10 induced elevation of CD107a on NK cells that were either co-cultured with K562 cells (Figures 6E,F) or stimulated with anti-CD16 antibody (Figures 6G,H). Similarly, we found that rapamycin treatment led to a decrease in IL-10-augmented granzyme B secretion in NK cells treated with anti-CD16 antibody (Figure 6I). It was also evident that rapamycin inhibited NK cell effector functions in a dose-dependent manner. We found that rapamycin started to show inhibitory effects on NK cell cytotoxicity at a concentration of 5nM (Figure 6J). The inhibitory effects increased as the concentration of rapamycin rose from 5 to 50 nM. Similarly, granzyme B (Figure 6K) and IFN-γ secretion (Figure 6L) of IL-10 stimulated NK cells also decreased as rapamycin concentration increases. Together, our results above indicate that mTORC1 activity is required for the enhanced effector functions of NK cells stimulated with IL-10.

## Discussion

Our current study showed that IL-10 treatment increases NK cell production of IFN-γ and enhances their cytotoxicity by augmenting their degranulation and FasL expression. Furthermore, we demonstrated that IL-10 boosts glycolysis and OXPHOS of NK cells, and these metabolic changes are required for the enhanced effector functions of IL-10 stimulated NK cells. Mechanistically, IL-10 stimulation triggers the activation of mTORC1 signaling, which is essential for the IL-10-induced metabolic reprogramming and enhanced effector functions of NK cells.

As the founding member of the IL-10 cytokine family, IL-10 exerts either stimulatory or inhibitory effects on various immune cells (13). These effects are elicited upon the binding of IL-10 to IL-10R that comprises α and β chains, with the α chain specifically binding to IL-10 with high affinity (10, 13). Here, we demonstrated that both *ex vivo* expanded and freshly-isolated human NK cells express IL-10Rα. The serum level of IL-10 in healthy adults is usually low, but it can be significantly elevated in patients with cancer or autoimmune diseases (37–39). For example, serum concentration of IL-10 in patients with malignant melanoma can reach as high as 23 ng/ml (39). As IL-10 has a short half-life and its effectiveness is constrained by distance (40), it could be found in higher concentration in tumor microenvironment or inflammation site than in the serum. In our study, we determined the effect of different concentrations of IL-10 on NK cells. We found that 5 ng/ml of IL-10 only slightly increased NK cell cytotoxicity and IFN-γ production, which is similar to previous research (23). However, IL-10 treatment at a higher concentration (50 ng/ml) has the optimal stimulatory effects on NK cells. It significantly enhanced the cytotoxicity of freshly-isolated human NK cells and increased IFN-γ production and cytotoxicity of *ex vivo* expanded NK cells. This finding is consistent with the results shown in another study demonstrating that IL-10 (30 ng/ml) increased NK cell killing of Daudi cells and increased their expression of activation and cytotoxicity related genes, such as perforin, CD16, TIA-1 and HMG-1 (20). We further showed that IL-10 treatment activated granule exocytosis in NK cells, as evidenced by their heightened expression of CD107a and granzyme B. We found that IL-10 promoted IFN-γ production and cytotoxicity of NK cells when they were co-stimulated with anti-CD16 antibody or co-cultured with K562 target cells, confirming the stimulatory roles of IL-10 on human NK cells.

It is known that the treatment of cytokines (e.g., IL-2, IL-12/IL-15) or the engagement of various NKRs can induce metabolic reprogramming in NK cells (9, 26–28, 35). However, it remained unclear if IL-10 could affect the metabolism of NK cells, as unlike these stimulatory cytokines, IL-10 has both inflammatory and anti-inflammatory properties. Our current study demonstrated that IL-10 induces metabolic reprogramming in both *ex vivo* expanded and freshly-isolated NK cells by upregulating their glycolysis and OXPHOS. More importantly, these metabolic changes are required for the enhanced effector functions of NK cells upon IL-10 treatment. Our metabolism results also indicate that freshly-isolated and *ex vivo* expanded NK cells are likely to have different metabolic dependence. Specifically, freshly-isolated NK cells with or without IL-10 stimulation had a low rate of glycolysis and relied more on OXPHOS for cell activity, while NK cells after expansion might shift their metabolic dependence to glycolysis. Glycolysis not only provides energy but also intermediates to generate biomass, e.g., ribose-5-phosphate for synthesizing the ribose of nucleotides and erythrose 4-phosphate for producing amino acids, which is critical for cell expansion (41). A recent study showed that IL-10 suppressed LPS-induced activation of macrophages by abrogating the enhanced glycolysis induced by LPS (30). The different effects of IL-10 on different cell types and in different stimulation settings suggest that IL-10 might have cell type- and activation-specific effects on cell metabolism.

Another interesting finding of our study is that IL-10 activates mTORC1 signaling in NK cells. It has been shown that the mTORC1 is activated in human NK cells stimulated by IL-2 and supports IL-2-induced glycolysis (27). On the other hand, mTORC1 activity is not required for the increased glycolysis of NK cells stimulated by IL-12 and IL-15 (27), suggesting that the role of mTORC1 in NK cell metabolism is cytokine-dependent. We found that inhibiting mTORC1 by rapamycin significantly suppressed the enhanced glycolysis and OXPHOS and effector functions of IL-10-treated NK cells, indicating that mTORC1 signaling is essential for the metabolic reprogramming and enhanced effector functions of IL-10 stimulated NK cells. We further showed that the activation of mTORC1 is downstream of STAT3 activation, which occurs upon IL-10R engagement. Inhibiting STAT3 suppresses IL-10-induced mTORC1 activation, but inhibiting mTORC1 by rapamycin has little effect on the activation of STAT3. However, the stimulatory role of IL-10 in NK cells shown in our study is different from its inhibitory role in macrophages. Ip et al. previously showed that IL-10 signaling via STAT3 suppressed glucose uptake of LPS-activated macrophages via dampening mTORC1 activation (30). They demonstrated that IL-10 induced the expression of DDIT4, an inhibitor of mTOR pathway, in macrophages via activation of STAT3. It is interesting to understand why IL-10 exhibits opposite effects on mTORC1 and glycolysis in macrophages and NK cells. We speculate that the basal activation state of mTORC1 might differ in the macrophages and NK cells of the two studies; therefore, IL-10 modulates mTOCR1 activity differently in these two types of cells. NK cells express much higher levels of DDIT4 than myeloid cells (https://www.proteinatlas.org/ENSG00000168209-DDIT4/blood), implying a lower basal activation of mTOCR1 constrained by DDIT4 in NK cells. Also, Ip et al. pre-stimulated the macrophages with LPS, which is known to activate mTORC1 in many cell types, including macrophages (42–44). Therefore, LPS stimulated macrophages may have a higher level of mTORC1 activity than NK cells before IL-10 treatment.

Collectively, our study suggests that IL-10 enhances NK cell effector functions by triggering metabolic reprogramming. We also demonstrate that mTORC1 is activated by IL-10 and is required to support the metabolic reprogramming and the enhanced effector functions of NK cells.

## Data Availability Statement

The original contributions presented in the study are included in the article/Supplementary Material, further inquiries can be directed to the corresponding author/s.

## Ethics Statement

The studies involving human participants were reviewed and approved by Institutional Review Board of Singapore Immunology Network (SIgN). The patients/participants provided their written informed consent to participate in this study.

## Author Contributions

ZW, SX, and K-PL designed the work and wrote the manuscript. ZW, DG, JH, and YH conducted the experiments. YY and SB provided critical reagents. All authors contributed to the article and approved the submitted version.

## Conflict of Interest

The authors declare that the research was conducted in the absence of any commercial or financial relationships that could be construed as a potential conflict of interest.
