# Supplementary material for: IL-10 Enhances Human Natural Killer Cell Effector Functions via Metabolic Reprogramming Regulated by mTORC1 Signaling
Source: Front Immunol. 2021 Feb 23;12:619195. doi: 10.3389/fimmu.2021.619195 (PMC7940510; doi:10.3389/fimmu.2021.619195)
Supplement: Supplementary file 1 [file Data_Sheet_1.PDF]

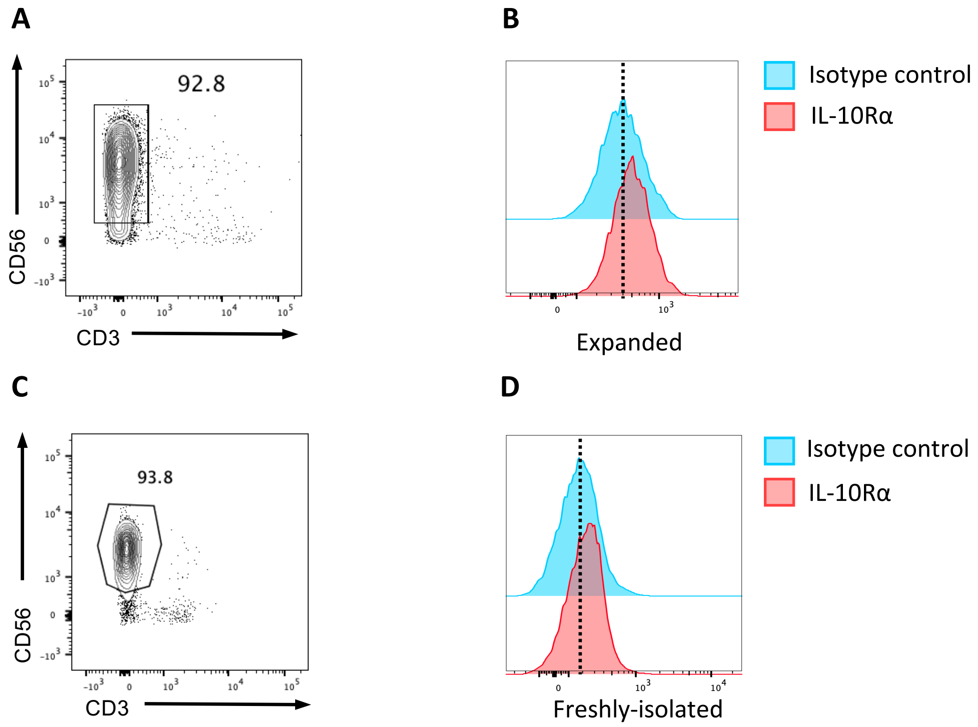

Figure S1. The expression of IL-10R α chain on *ex vivo* expanded and freshly-isolated NK cells. (**A**) The purity of NK cells after *ex vivo* expansion. (**B**) The expression of IL-10R α chain (IL-10Rα) on *ex vivo* expanded NK cells was examined by flow cytometry. (**C**) The purity of NK cells freshly isolated from human PBMCs. (**D**) The expression of IL-10Rα on freshly-isolated NK cells was examined by flow cytometry.

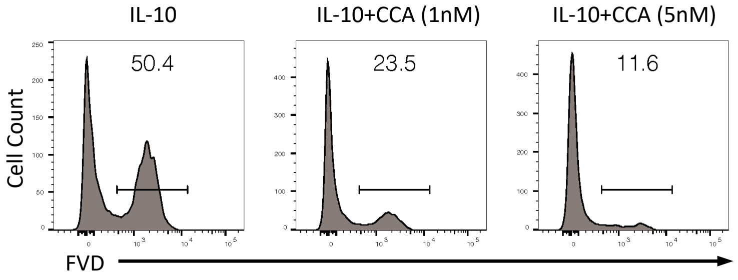

Figure S2. Inhibiting degranulation impaired the cytotoxicity of IL-10 stimulated NK cells. *Ex vivo* expanded NK cells were stimulated with IL-10 for 16h before treated with 1nM or 5nM of concanamycin A (CCA) for 3h. The pre-treated NK cells were washed with PBS twice and co-cultured with CellTrace violet labeled-K562 cells for 1.5h at E:T ratio of 1.5:1. Dead K562 cells were determined by flow cytometry analysis of cells stained with FVD, and the percentages of dead K562 cells were shown in the histogram.

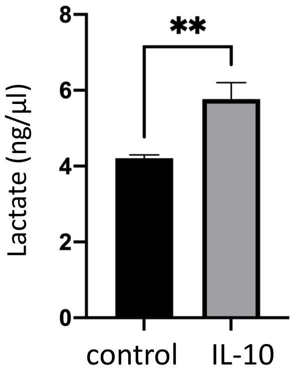

Figure S3. The production of lactate by NK cells with or without IL-10 stimulation. *Ex vivo* expanded NK cells were stimulated with IL-10 for 16h or left unstimulated. The supernatant was collected and assayed for lactate detection. Data were presented as Mean ± SD (n=3) and compared using independent Student's t-test. **, P<0.01.

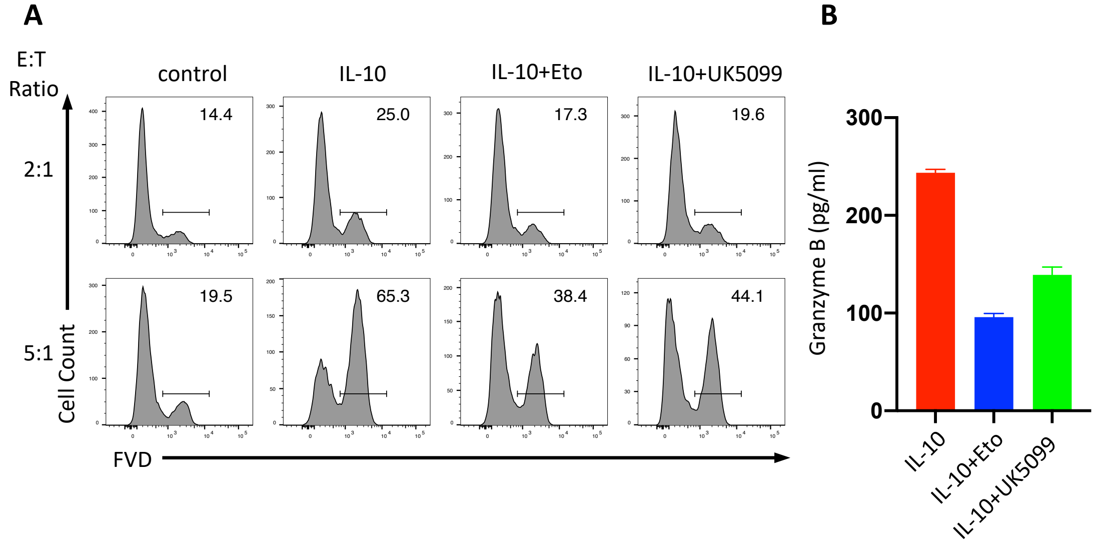

Figure S4. Fatty acid oxidation and glycolysis contribute to the cytotoxicity of IL-10 stimulated freshly-isolated NK cells. (A) Killing of K562 cells by IL-10 stimulated NK cells treated with or without etomoxir or UK5099. Freshly isolated NK cells were stimulated with IL-10 for 16h before treated with etomoxir (Eto, 150μM) or UK5099 (10μM) for 3h. The pre-treated NK cells were washed with PBS twice before co-cultured with CellTrace violet labeled-K562 cells for 3h at E:T ratios of 2:1 and 5:1. Dead K562 cells were determined by flow cytometry analysis of cells stained with FVD. Unstimulated NK cells that co-cultured with K562 cells served as control. (**B**) The granzyme B secretion of IL-10 stimulated NK cells with or without etomoxir or UK5099 treatment. NK cells were stimulated with IL-10 for 6h and treated as described in (**A**) for 6h. The supernatant was collected and granzyme B was detected using ELISA.
